# High‐Intensity Focused Ultrasound for Botulinum Toxin‐Induced Ptosis: A Retrospective Pilot Case Series

**DOI:** 10.1111/jocd.71109

**Published:** 2026-07-28

**Authors:** Mojtaba Nasimi, Farideh Nasiri

**Affiliations:** ^1^ Department of Dermatology Birjand University of Medical Sciences Birjand Iran; ^2^ Birjand University of Medical Sciences Birjand Iran

**Keywords:** botulinum toxin, Dysport, HIFU, high‐intensity focused ultrasound, MRD1: Oxymethazoline, ptosis

## Abstract

**Background:**

Botulinum toxin‐induced ptosis is a recognized complication of aesthetic injections that may adversely affect cosmetic outcomes and patient satisfaction. Pharmacologic management is limited and primarily aims to provide temporary elevation of the upper eyelid through stimulation of Müller's muscle. High‐intensity focused ultrasound (HIFU) has established applications in facial tissue tightening and may represent a potential non‐pharmacologic treatment option.

**Objective:**

To evaluate the clinical outcomes of botulinum toxin‐induced ptosis treated with HIFU using the ULTRAcel Q+ system.

**Methods:**

This retrospective pilot case series included five consecutive patients with botulinum toxin‐induced ptosis treated using a standardized HIFU protocol with the ULTRAcel Q+ system (3‐mm dot handpiece, energy level 0.7, 12 shots). Clinical outcomes were evaluated using standardized photographs obtained under controlled imaging conditions. Margin Reflex Distance 1 (MRD1), defined as the distance between the corneal light reflex and the upper eyelid margin in primary gaze. Eyebrow position was also evaluated by comparing brow height relative to the supraorbital rim and facial symmetry. Both were measured at baseline, immediately after treatment, and during follow‐up through Day 21.

**Results:**

Five patients (4 females, 1 male; mean age 38.4 years) were included. All had received Dysport injections (130–150 units) to the glabella, forehead, and crow's feet regions. Ptosis onset occurred 3–7 days after injection. HIFU was performed 3–6 days after symptom onset. Mean MRD1 increased from 0.76 mm at baseline to 1.98 mm immediately after treatment, 2.30 mm at Day 7, 4.18 mm at Day 14, and 4.44 mm at Day 21. Three patients presented with isolated eyelid ptosis, whereas two had combined eyelid and eyebrow ptosis. All patients demonstrated complete clinical resolution by Day 14. Mild transient procedural pain occurred during treatment, and no long‐term treatment‐related adverse events were observed.

**Conclusion:**

In this retrospective pilot case series, HIFU using the ULTRAcel Q+ system was associated with complete clinical resolution of botulinum toxin‐induced ptosis by Day 14 and was well tolerated. Unlike pharmacologic agents such as oxymetazoline, which temporarily elevate the eyelid through Müller's muscle stimulation, HIFU may act through a different mechanism involving tissue contraction and remodeling; however, the contribution of spontaneous recovery cannot be excluded. Larger prospective controlled studies are needed to determine efficacy and clarify the underlying mechanisms.

## 1 Introduction

Botulinum toxin type A is widely used in aesthetic medicine for the treatment of dynamic facial rhytides because of its favorable efficacy and safety profile. Despite its widespread use, unintended diffusion of the toxin into adjacent muscles may result in complications, including eyelid ptosis and less commonly, eyebrow ptosis. These complications are generally transient but can cause functional impairment, cosmetic dissatisfaction, and psychological distress, often persisting for several weeks until neuromuscular transmission recovers naturally [1, 2].

Current management of botulinum toxin‐induced blepharoptosis is primarily conservative. Oxymetazoline hydrochloride 0.1% ophthalmic solution is currently the only U.S. Food and Drug Administration (FDA)‐approved pharmacologic treatment for acquired blepharoptosis and has also been proposed as a therapeutic option for botulinum toxin‐induced ptosis. Off‐label apraclonidine has historically been used to provide temporary eyelid elevation through stimulation of Müller's muscle. However, these medications offer symptomatic improvement rather than reversal of the underlying chemodenervation and require repeated administration until spontaneous recovery occurs [3, 4].

High‐intensity focused ultrasound (HIFU) is a well‐established noninvasive technology used for facial rejuvenation and tissue tightening. By creating focal thermal coagulation zones at precise tissue depths, HIFU induces immediate collagen contraction followed by neocollagenesis and tissue remodeling, resulting in progressive lifting and tightening of the treated tissues [5, 6]. Although these biological effects have been extensively investigated for aesthetic indications, their potential role in the management of botulinum toxin‐induced ptosis has not been studied.

The mechanism by which HIFU might improve botulinum toxin‐induced ptosis remains speculative. Immediate tissue contraction and subsequent remodeling of the periocular soft tissues could potentially contribute to improved eyelid and eyebrow position independently of neuromuscular recovery. Whether these structural effects translate into clinically meaningful improvement in botulinum toxin‐induced ptosis has not been previously reported.

The present retrospective pilot case series evaluated the clinical outcomes of botulinum toxin‐induced ptosis treated with a standardized HIFU protocol using the ULTRAcel Q+ system. Clinical outcomes were assessed using standardized photographic analysis of Margin Reflex Distance 1 (MRD1) and eyebrow position to provide preliminary evidence regarding the feasibility, safety, and potential therapeutic role of HIFU for this complication.

## 2 Materials and Methods

### 2.1 Study Design and Patients

This retrospective pilot case series included five consecutive patients who developed botulinum toxin‐induced ptosis following cosmetic treatment with abobotulinumtoxinA (Dysport) in routine clinical practice. Medical records and standardized clinical photographs were retrospectively reviewed. All patients had received their first botulinum toxin treatment, with total doses ranging from 130 to 150 units administered to the glabellar, forehead, and lateral canthal (crow's feet) regions.

Patients were eligible if they developed unilateral or bilateral eyelid ptosis, with or without eyebrow ptosis, after botulinum toxin injection and subsequently underwent HIFU treatment using a standardized treatment protocol. Patients with pre‐existing ptosis, previous eyelid or brow surgery, neuromuscular disorders, or incomplete clinical documentation were excluded.

### 2.2 HIFU Treatment Protocol

**FIGURE 1:**
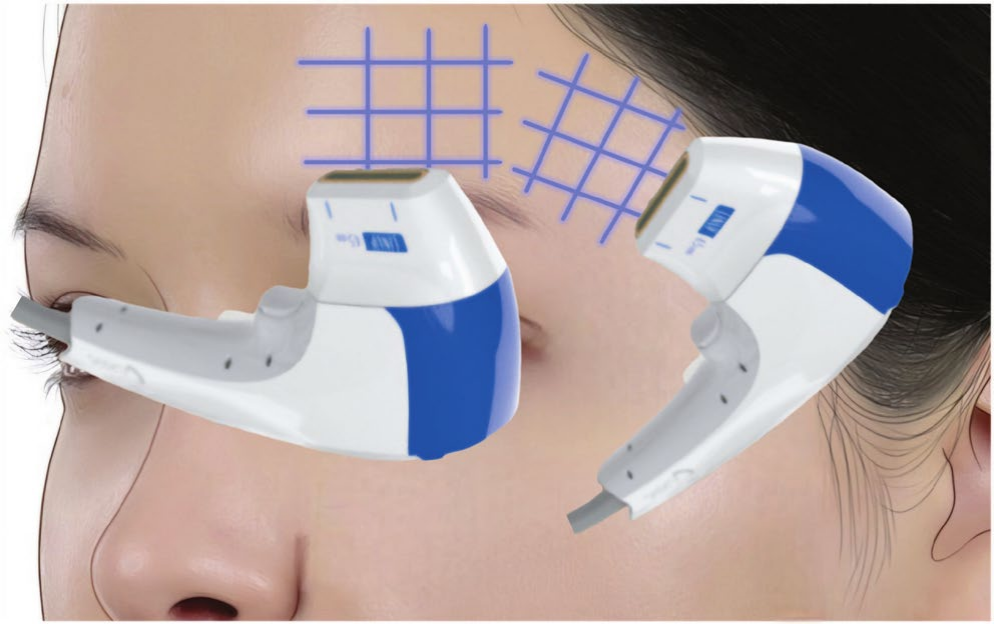
Schematic illustration of the standardized ULTRAcel Q+ treatment protocol over the supraorbital and frontal regions.

All patients underwent treatment using the ULTRAcel Q+ system (Jeisys Medical Inc., Seoul, Republic of Korea). A standardized treatment protocol was applied in every case using a 3‐mm dot transducer at an energy level of 0.7 J with 12 treatment shots. The treatment area and application pattern over the supraorbital and frontal regions were identical for all patients and are illustrated in Figure 1. No additional interventions for ptosis, including topical pharmacologic agents, were administered during the observation period.

### 2.3 Clinical Outcome Assessment

Clinical outcomes were evaluated using standardized digital photographs obtained before treatment, immediately after HIFU, and on Days 3, 7, 14, and 21 after treatment. Photographs were acquired under standardized conditions, including primary gaze, neutral facial expression, fixed camera position, constant camera‐to‐patient distance, identical lighting conditions, and consistent image magnification.

The primary outcome measure was Margin Reflex Distance 1 (MRD1), defined as the vertical distance between the corneal light reflex and the upper eyelid margin in primary gaze, measured according to the standard ophthalmic method described by Bartley and Bullock [7]. MRD1 measurements were obtained from standardized photographs.

Eyebrow position was evaluated qualitatively by comparing brow height relative to the supraorbital rim and by assessing symmetry between the treated and contralateral sides. Patients presenting with combined eyelid and eyebrow ptosis were assessed for improvement in both parameters.

Complete clinical resolution was defined as fulfillment of all of the following criteria:

- normalization of MRD1 compared with the unaffected eye or expected anatomical position;
- restoration of eyebrow position in patients with associated eyebrow ptosis;
- absence of clinically detectable ptosis on physical examination; and
- patient‐reported satisfaction with eyelid appearance.

### 2.4 Statistical Analysis

Given the small sample size, statistical analysis was descriptive. Continuous variables are presented as mean ± standard deviation (SD) and range, whereas categorical variables are summarized as frequencies and percentages. No inferential statistical analyses were performed because of the exploratory nature of this pilot study.

### 2.5 Ethics Statement

Written informed consent for treatment was obtained from all patients. Consent for publication of identifiable clinical photographs was not available; therefore, no patient photographs are included in this manuscript.

## 3 Results

### 3.1 Patient Characteristics

Five consecutive patients with botulinum toxin‐induced ptosis were included in the study, comprising four females and one male with a mean age of 38.4 years (range, 32–45 years). All patients developed unilateral ptosis following their first cosmetic treatment with abobotulinumtoxinA (Dysport). Total injected doses ranged from 130 to 150 units and were administered to the glabellar, forehead, and lateral canthal (crow's feet) regions.

The onset of ptosis occurred 3–7 days after botulinum toxin injection (mean ± SD, 4.8 ± 1.5 days). HIFU treatment was performed 3–6 days after symptom onset (mean ± SD, 4.2 ± 1.3 days). Three patients (H1, H3, and H5) presented with isolated eyelid ptosis, whereas two patients (H2 and H4) exhibited combined eyelid and eyebrow ptosis. No other asymmetry was observed. Baseline patient characteristics are summarized in Table 1.

**TABLE 1:** Patient characteristics.

Patient	Age (years)	sex	Ptosis onset^a^	HIFU application^b^	Ptosis side	Eyebrow ptosis
H1	38	F	3	3	Left	+
H2	40	F	5	4	Left	−
H3	32	M	7	3	Right	+
H4	45	F	4	5	Right	−
H5	37	F	5	6	Left	−

^a^ Days between Dysport injection and ptosis occurrence.

^b^ The interval between symptom onset and HIFU treatment.

### 3.2 Clinical Outcomes

Serial MRD1 measurements are presented in Table 2. The mean MRD1 increased progressively from 0.76 mm at baseline to 1.98 mm immediately after HIFU treatment, 1.78 mm on Day 3, 2.30 mm on Day 7, 4.18 mm on Day 14, and 4.44 mm on Day 21.

**TABLE 2:** Margin reflex distance 1 (MRD1) measurements following HIFU treatment.

Patient	Baseline (mm)	Immediate Post‐Treatment (mm)	Day 3 (mm)	Day 7 (mm)	Day 14 (mm)	Day 21 (mm)
H1	0.8	2.1	1.8	2.5	4.0	4.2
H2	0.5	2.0	1.7	2.0	4.2	4.5
H3	0.8	1.5	1.9	2.5	4.7	5.0
H4	0.6	1.8	1.5	2.0	4.0	4.3
H5	1.1	2.5	2.0	2.5	4.0	4.2
Mean	0.76	1.98	1.78	2.30	4.18	4.4

Abbreviation: MRD1, margin reflex distance 1.

An immediate increase in MRD1 was observed in all patients following HIFU treatment. Progressive improvement continued throughout the follow‐up period, with the greatest increase occurring between Days 7 and 14. By Day 14, all patients had achieved normalization of eyelid position based on clinical examination and MRD1 assessment. These improvements were maintained at the Day 21 follow‐up.

Among the two patients with combined eyelid and eyebrow ptosis, both eyelid position and eyebrow symmetry improved progressively after treatment, with complete restoration of eyebrow position observed by Day 14.

**FIGURE 2:**
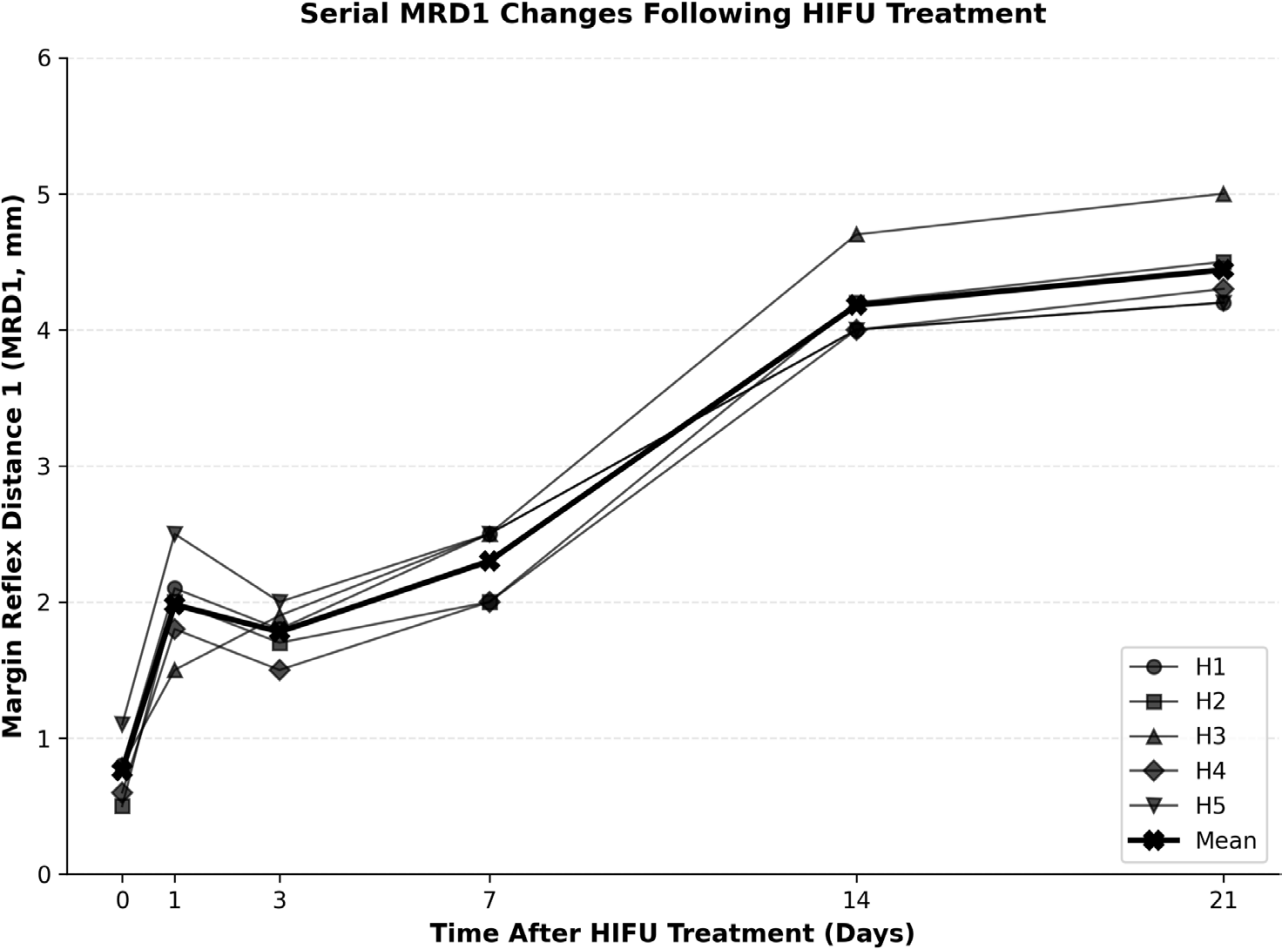
Serial MRD1 changes over time following HIFU treatment in five patients with botulinum toxin‐induced ptosis.

**FIGURE 3:**
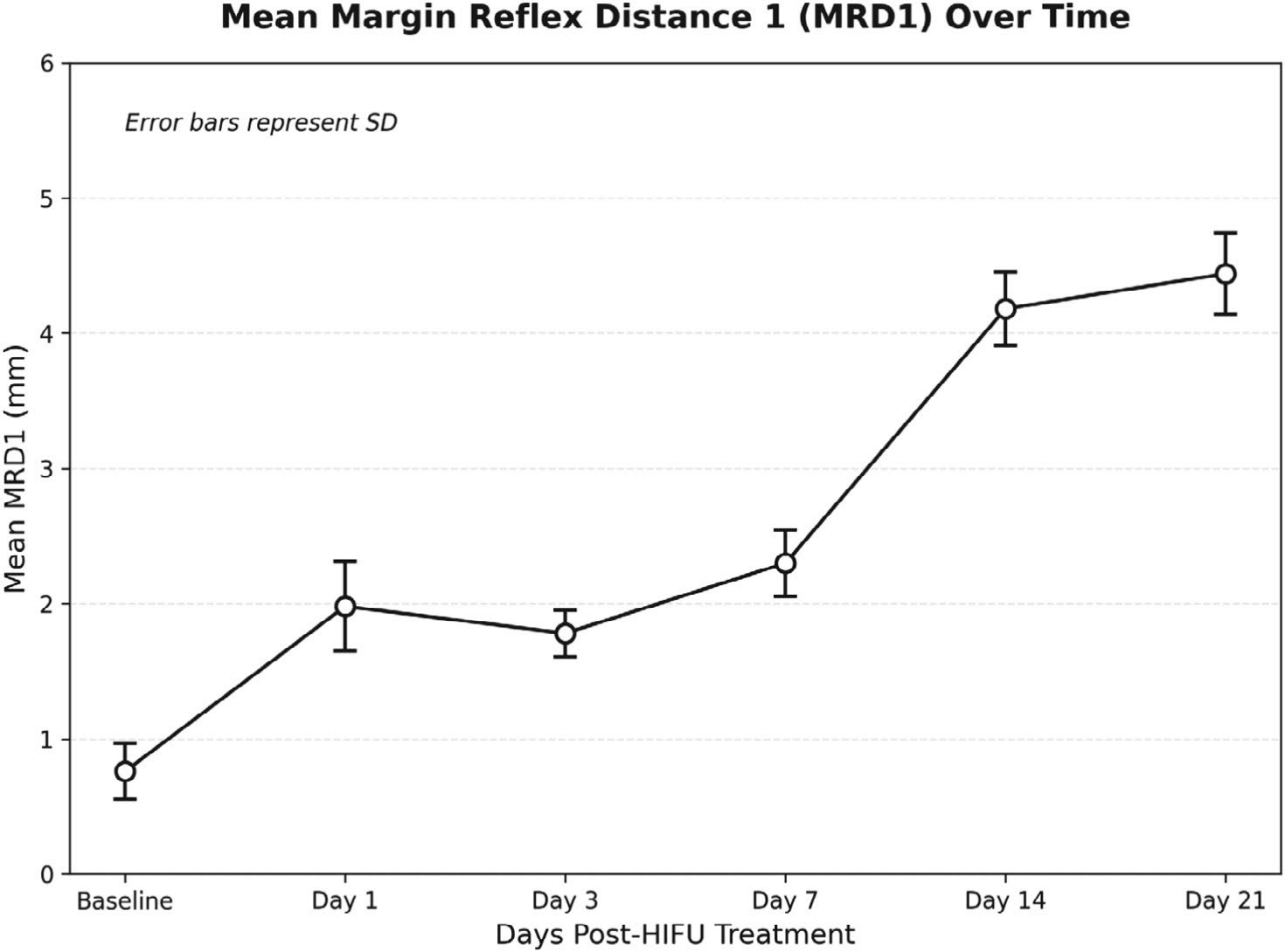
Clinical timeline and recovery trend demonstrating progressive resolution of ptosis by Day 14.

The temporal changes in MRD1 are illustrated in Figure 2, and the overall clinical recovery pattern is summarized in Figure 3.

### 3.3 Safety

All patients completed the HIFU procedure without interruption. Mild transient procedural pain during energy delivery was reported by all patients but did not require treatment or discontinuation of the procedure. No immediate or delayed treatment‐related adverse events, including burns, blistering, scarring, visual disturbances, infection, or worsening of ptosis, were observed during the 21‐day follow‐up period.

## 4 Discussion

This retrospective pilot case series evaluated the clinical outcomes of botulinum toxin‐induced ptosis treated with a standardized HIFU protocol using the ULTRAcel Q+ system. All five patients demonstrated progressive improvement in MRD1 and complete clinical resolution by Day 14 without long‐term treatment‐related adverse events. Improvements were observed immediately after HIFU treatment and continued throughout the follow‐up period, with the greatest increase in MRD1 occurring between Days 7 and 14.

Botulinum toxin‐induced ptosis results from unintended diffusion of the toxin into the levator palpebrae superioris muscle, producing temporary chemodenervation and reduced upper eyelid elevation. Symptoms typically develop within several days after injection and gradually resolve as neuromuscular transmission recovers through axonal sprouting and regeneration of functional neuromuscular junctions. Although spontaneous recovery is expected, complete resolution commonly requires several weeks, during which patients may experience functional impairment and cosmetic dissatisfaction [1, 2, 8].

Current treatment of botulinum toxin‐induced blepharoptosis is primarily symptomatic. Oxymetazoline hydrochloride 0.1% ophthalmic solution is the only FDA‐approved medication for acquired blepharoptosis and has been proposed as an effective treatment for botulinum toxin‐induced ptosis through stimulation of α‐adrenergic receptors in Müller’s muscle, producing temporary eyelid elevation. Apraclonidine has also been used off‐label through a similar pharmacologic mechanism. Neither medication reverses the underlying chemodenervation of the levator palpebrae superioris muscle, and repeated administration is required until neuromuscular function recovers spontaneously [3, 4].

The mechanism by which HIFU may improve botulinum toxin‐induced ptosis is likely distinct from that of pharmacologic agents and remains speculative. HIFU delivers focused ultrasound energy that creates precise thermal coagulation zones within targeted tissues while sparing the overlying epidermis. These thermal effects produce immediate collagen contraction, followed by fibroblast activation, neocollagenesis, and progressive tissue remodeling over subsequent weeks. In the periocular region, these structural changes may increase soft‐tissue support and contribute to elevation of the upper eyelid and eyebrow independently of direct restoration of levator muscle function [5, 6].

The immediate increase in MRD1 observed following HIFU treatment may reflect acute tissue contraction and mechanical tightening of periocular connective tissues. Continued improvement between Days 7 and 14 is consistent with the early phase of tissue remodeling described after HIFU treatment. Nevertheless, the contribution of the natural decline in botulinum toxin activity cannot be excluded. Because this study lacked an untreated control group, it is not possible to determine the relative contribution of HIFU and spontaneous recovery to the observed clinical improvement.

To our knowledge, this is the first reported case series evaluating HIFU as a treatment for botulinum toxin‐induced ptosis. Unlike pharmacologic therapy, which provides temporary functional elevation through stimulation of Müller's muscle, HIFU may offer a non‐pharmacologic approach based on structural tissue effects. These differing mechanisms suggest that HIFU could represent a complementary therapeutic option, although comparative clinical studies are required to establish its efficacy relative to existing treatments.

This study has several limitations. The principal limitations include the retrospective design, small sample size, absence of an untreated or pharmacologic control group, and short follow‐up period. In addition, representative clinical photographs could not be included because consent for publication was not available. Although outcomes were assessed using standardized photographic acquisition and objective MRD1 measurements, the absence of published clinical images limits visual documentation of treatment response. Finally, the observational design precludes conclusions regarding causality.

Despite these limitations, the use of a standardized treatment protocol, objective MRD1 measurements obtained under controlled imaging conditions, and consistent follow‐up across all patients provides preliminary evidence supporting the feasibility and safety of HIFU for botulinum toxin‐induced ptosis. Larger prospective controlled studies comparing HIFU with observation alone and with pharmacologic treatments such as oxymetazoline are warranted to determine efficacy, define optimal treatment parameters, and further clarify the biological mechanisms underlying clinical improvement.

## 5 Conclusion

In this retrospective pilot case series, treatment with HIFU using the ULTRAcel Q+ system was associated with progressive improvement in eyelid position and complete clinical resolution of botulinum toxin‐induced ptosis by Day 14 in all patients. The procedure was well tolerated, with only mild transient procedural pain and no long‐term treatment‐related adverse events observed during follow‐up.

Although the findings suggest that HIFU may represent a promising non‐pharmacologic treatment option for botulinum toxin‐induced ptosis, the contribution of spontaneous recovery cannot be excluded because of the absence of a control group. Larger prospective controlled studies comparing HIFU with observation and established pharmacologic therapies, including oxymetazoline, are needed to determine efficacy, establish causality, optimize treatment protocols, and further elucidate the underlying mechanisms of action.

## Conflicts of Interest

The authors declare no conflicts of interest.

## Data Availability

Data are available upon reasonable request from the corresponding author.
